# Amplitude of Low-Frequency Fluctuation in Multiple Frequency Bands in Tension-Type Headache Patients: A Resting-State Functional Magnetic Resonance Imaging Study

**DOI:** 10.3389/fnins.2021.742973

**Published:** 2021-10-25

**Authors:** Meng-Ting Li, Shu-Xian Zhang, Xue Li, Collins Opoku Antwi, Jia-Wei Sun, Chao Wang, Xi-He Sun, Xi-Ze Jia, Jun Ren

**Affiliations:** ^1^School of Teacher Education, Zhejiang Normal University, Jinhua, China; ^2^Key Laboratory of Intelligent Education Technology and Application of Zhejiang Province, Zhejiang Normal University, jinhua, China; ^3^Department of Medical Imaging, Affiliated Hospital of Weifang Medical University, Weifang, China; ^4^School of Information and Electronics Technology, Jiamusi University, Jiamusi, China

**Keywords:** tension-type headache, amplitude of low-frequency fluctuation (ALFF), frequency band specificity, resting-state functional MRI, low-frequency oscillations (LFO)

## Abstract

**Purpose:** Tension-type headache (TTH), the most prevalent primary headache disorder, imposes an enormous burden on the people of the world. The quest to ease suffering from this neurological disorder has sustained research interest. The present study aimed at evaluating the amplitude of low-frequency oscillations (LFOs) of the brain in multiple frequency bands in patients with TTH.

**Methods:** To address this question, 63 participants were enrolled in the study, including 32 TTH patients and 31 healthy controls (HCs). For all the participants, amplitude of low-frequency fluctuation (ALFF) was measured in six frequency bands (conventional frequency bands, 0.01–0.08 Hz; slow-2, 0.198–0.25 Hz; slow-3, 0.073–0.198 Hz; slow-4, 0.027–0.073 Hz; slow-5, 0.01–0.027 Hz; and slow-6, 0–0.01 Hz), and the differences between TTH patients and HCs were examined. To explore the relationship between the altered ALFF brain regions in the six frequency bands and the Visual Analog Scale (VAS) score in the TTH patients, Pearson’s correlation analysis was performed.

**Results:** In all the six frequency bands, a decreased ALFF value was detected, and regions showing reduced ALFF values were mostly located in the middle frontal gyrus and superior gyrus. A frequency-dependent alternating characterization of intrinsic brain activity was found in the left caudate nucleus in the slow-2 band of 0.198–0.25 Hz and in the right inferior frontal orbital gyrus in the slow-5 band of 0.01–0.027 Hz. For the correlation results, both the left anterior cingulate and paracingulate gyri and right superior parietal gyrus showed a positive correlation with the VAS score in the slow-4 frequency band of 0.027–0.073 Hz.

**Conclusion:** The ALFF alterations in the brain regions of TTH patients are involved in pain processing. The altered LFOs in the multiple regions may help promote the understanding of the pathophysiology of TTH. These observations could also allow the future treatment of TTH to be more directional and targeted and could promote the development of TTH treatment.

## Introduction

Tension-type headache (TTH) inflicts a high degree of disability on individuals, limiting their productive capacities and debilitating overall quality of life (Bendtsen et al., 2010a). Up to 2016, the prevalent neurological disorder has affected 1.89 billion people worldwide (Stovner et al., 2018). Paradoxically, studies on the most frequent headache (i.e., TTH) are relatively scarce despite its prevalence and costs (Stovner et al., 2007). Moreover, there are no significant advances in the treatment of TTH in recent decades, which may be mainly due to the lack of in-depth understanding of its pathophysiology (Bendtsen et al., 2010b).

Recently, resting-state functional magnetic resonance imaging (rs-fMRI) based on the blood oxygenation level-dependent (BOLD) signal has been applied to measure spontaneous brain activity when individuals perform no task (Biswal et al., 1995; Fox et al., 2005; Fox and Raichle, 2007). Rs-fMRI has been applied in studies using patients with Parkinson’s disease (Bi et al., 2021), schizophrenia (Yang M. et al., 2018), and depression (Yu H. et al., 2020). Several approaches have been proposed to characterize the local properties of the rs-fMRI signal, such as amplitude of low-frequency fluctuation (ALFF; Yu-Feng et al., 2007) and regional homogeneity (ReHo; Zang et al., 2004). A considerable number of studies have utilized other techniques, including ReHo, in teasing out resting-state brain activities in TTH patients. For instance, Wang et al. (2014) investigated the regional synchronizations of temporal changes in BOLD activity in TTH patients using the ReHo analysis. They found lower ReHo values in the bilateral caudate nucleus, the left middle frontal gyrus, and the superior frontal gyrus. However, TTH studies employing the ALFF method are relatively sparse. ALFF is a resting-state data analysis method that measures the BOLD signal fluctuations within the gray matter (GM) and reflects the local properties of spontaneous neuronal activity (Yu-Feng et al., 2007; Zou et al., 2008; Zhang et al., 2010). Though the use of ALFF in TTH studies is limited, it has been widely applied to detect changes in regional signals in patients with bipolar disorder (Zhang et al., 2020), schizophrenia (Wang et al., 2019), Alzheimer’s disease (Yang L. et al., 2018), and migraine (Wang et al., 2016a). These studies’ results demonstrate the promise of ALFF as a powerful method for TTH research.

Therefore, the current study aimed at examining the regional spontaneous neuronal activity of TTH patients during resting state with ALFF. We believe that the application of the ALFF approach could improve our understanding of the TTH. Most studies about ALFF focused on conventional frequency bands, that is, 0.01–0.08 Hz (Wang et al., 2018; Luo et al., 2020). Yu J. et al. (2020) found the results from one frequency band as lacking in frequency characteristics. Previous studies illustrated frequency bands as concerned with neuronal undulations. In this respect, the inherent neuronal oscillation patterns in the brain are very sensitive to specific frequency bands (Buzsaki, 2004; Gao et al., 2015). Therefore, different oscillation frequencies show specific peculiarities and are involved in different aspects of brain functions (Egorova et al., 2017; Yu J. et al., 2020). The frequency spectrum was typically subdivided into five different frequency bands: slow-6 (0–0.01 Hz), slow-5 (0.01–0.027 Hz), slow-4 (0.027–0.073 Hz), slow-3 (0.073–0.198 Hz), and slow-2 (0.198–0.25 Hz) (Buzsaki, 2004; Zuo et al., 2010). Although most studies discarded the very low-frequency band <0.01 Hz (slow-6), slow-6 was indicated to be meaningful in either physiological (Lv et al., 2013; Zhang H. et al., 2015) or pathophysiological (Wang et al., 2015) studies. The frequency-dependent effects of ALFF are investigated in plenty of neurological and psychiatry disorders, including schizophrenia (Qi et al., 2018), chronic obstructive pulmonary disease (Yu J. et al., 2020), depression (Egorova et al., 2017), and social anxiety disorder (Zhang Y. et al., 2015). However, studies about frequency-specific changes of ALFF in TTH are lacking.

In the present work, we explore the local spontaneous activity in TTH patients. Meanwhile, a frequency-dependent analysis was conducted to identify the abnormalities in spontaneous fluctuations in TTH. Based on the previous studies, we hypothesized that altered ALFF in TTH patients might be related to specific frequency bands.

## Materials and Methods

### Subjects

Data were obtained from 38 right-handed TTH patients from the outpatient clinic of the Affiliated Hospital of Weifang Medical University. The diagnostic criteria of TTH are based on the International Classification of Headache Disorders 3rd Edition, beta version criteria (ICHD-3) (Headache Classification Committee of the International Headache Society HIS, 2013). Two neurologists made a clinical diagnosis that satisfied the following headache characteristics: bilateral, mild-to-moderate intensity, non-pulsating, and not aggravated by routine physical activity. All patients underwent the Visual Analog Scale (VAS) evaluation, which evaluates the subjective severity and relief of pain or discomfort (Kelly, 2001), and we collected their demographic information, including age, sex and, education, during the interview. We recruited 38 healthy controls (HCs) who recorded no history of headaches. All participants’ age ranged from 18 to 60 years, and none had a history of neurological and psychiatric illness. Exclusion criteria were as follows: (1) alcohol and drug abuse; (2) suffering from other types of headache or chronic pain disorders; (3) intracranial lesions in previous MRI or CT scans; (4) pregnancy or menstrual period in women; and (5) claustrophobia. Five TTH patients were excluded according to the criteria above.

The present study was approved by the Affiliated Hospital of Weifang Medical University Committee on Human Research with 2021YX118 registration code. Each participant provided a written informed consent.

### Magnetic Resonance Imaging Acquisition

Magnetic resonance imaging scanning was performed on a 3.0-T MRI scanning system (Signa HDxt, GE Medical Systems, Chicago, IL, United States) with an eight-channel phase array head coil. Plastic foam pads were applied to minimize head movement, and two appropriately sized earplugs diminished scanner noise. All participants were required to remain still with their eyes closed but without pondering or sleeping during the whole scanning process. The scanning was terminated if the participant showed any discomfort.

First, T2-weighted imaging (T_2_WI) was performed in all subjects to exclude the possibility of asymptomatic lesions.

Resting-state functional magnetic resonance imaging data were obtained using an echo-planar imaging sequence with the following parameters: repetition time (TR) = 2,000 ms, echo time (TE) = 30 ms, flip angle = 90°, slice thickness = 4.0 mm, matrix = 64 × 64, field of view (FOV) = 240 mm × 240 mm, number of slices = 32, and total volume = 200. The session lasted 400 s.

Three-dimensional high-resolution T1-weighted anatomical images were acquired using the spoiled gradient recalled acquisition, TR = 7.8 ms, TE = 3.0 ms, flip angle = 15°, slice thickness = 1.0 mm, FOV = 256 mm × 256 mm, matrix = 256 × 256, and number of slices = 188; the session lasted 250 s.

### Data Preprocessing

Resting-state functional magnetic resonance imaging data were processed using *RESTplus* V1.24 (Jia et al., 2019). The first 10 time points were discarded to overcome the instability of the initial MRI signal and to ensure that the participants get used to the scanner noise. Second, a slice-timing correction was conducted to correct the differences in the acquisition time. Third, head motion correction was performed. Fourth, the individual structural image was co-registered to the mean functional image. Then, the co-registered structural images were segmented into the GM, white matter (WM), cerebrospinal fluid (CSF), bone, soft tissue, and air/background. Finally, spatial normalization into the Montreal Neurological Institute (MNI) space was computed with Diffeomorphic Anatomical Registration Through Exponentiated Lie algebra tool (DARTEL, Ashburner, 2007) and resampled at 3 mm × 3 mm × 3 mm. Fifth, spatial smoothing *via* a Gaussian kernel with full width at half maximum (FWHM) = 4 mm. Sixth, the linear trend of the time course was removed. Next, to remove the influence of global signal, the global mean signal was regressed out from the fMRI data (Macey et al., 2004; Fox et al., 2009). In addition, the WM signal was removed to reduce respiratory and cardiac effects (Chen et al., 2018). To remove the confound of head motion, the 24 head motion parameters were regressed out (Friston et al., 1996; Satterthwaite et al., 2012; Zeng et al., 2014). Eight participants with a maximum translation in any direction >2.5 mm and a maximum rotation >2.5° were excluded from further analysis, which is consistent with previous studies (Yin, 2016; Bernier, 2017; Qiu et al., 2018).

### Amplitude of Low-Frequency Fluctuation Calculation

Five frequency bands (slow-2–slow-6) of ALFF values were calculated besides the conventional frequency band (0.01–0.08 Hz) for each participant. Specifically, the preprocessed time series were converted to a frequency domain using a fast Fourier transform (FFT), and the power spectrum was acquired. Then, the square root of each frequency of the power spectrum and the averaged square root of the frequency range we had predefined were taken as the ALFF values. For standardization, the ALFF value of each voxel was divided by the global mean ALFF value (Yu-Feng et al., 2007).

### Statistical Analysis

A two-sample *t*-test was conducted in Data Processing & Analysis for Brain Imaging (DPABI, V4.0) (Yan et al., 2016) to compare the ALFF differences between the TTH group and HC group. Age and frame-wise displacement (FD) parameters were regressed out in the statistical analysis. And the multiple comparison correction was based on the Threshold-Free Cluster Enhancement (TFCE; Smith and Nichols, 2009). Our TFCE significance threshold was set at *p*_FDR_ < 0.01. The number of permutations was set at 5,000.

### Correlation Analysis Between Amplitude of Low-Frequency Fluctuation and Visual Analog Scale Score

To explore the detailed associations between ALFF values and VAS scores in all the six frequency bands, the correlation analysis was conducted. Specifically, the brain regions showing significant differences between the TTH and HCs were defined as regions of interest (ROIs). Then, the mean ALFF value in every ROI was extracted, and Pearson’s correlation coefficient was calculated between the mean ALFF value and VAS score.

## Results

### Demographic Characteristics

The demographic characteristics of the TTH and HC groups are shown in Table 1. No significant differences in sex, education, and FD were observed between TTH and HCs. Five TTH patients were excluded due to being overage, and seven HCs and one TTH were excluded for excessive head motion as described above.

**TABLE 1:** Demographic characteristics of all participants.

	TTH	HCs	*p*-Value
	(*n* = 32)	(*n* = 31)	
Age (years)	42.78 ± 12.10	36.87 ± 10.01	0.039
Sex (M/F)	13/19	14/17	0.716
Education	10.28 ± 3.32	11.03 ± 2.77	0.335
VAS score	4.78 ± 1.29	–	–
FD (Jenkinson)	0.086 ± 0.3647	0.08 ± 0.03	0.258

*FD = frame-wise displacement for in-scanner head motion. TTH, tension-type headache; HCs, healthy controls; M, male; F, female.*

### Amplitude of Low-Frequency Fluctuation Analysis in Different Frequency Bands

**FIGURE 1:**
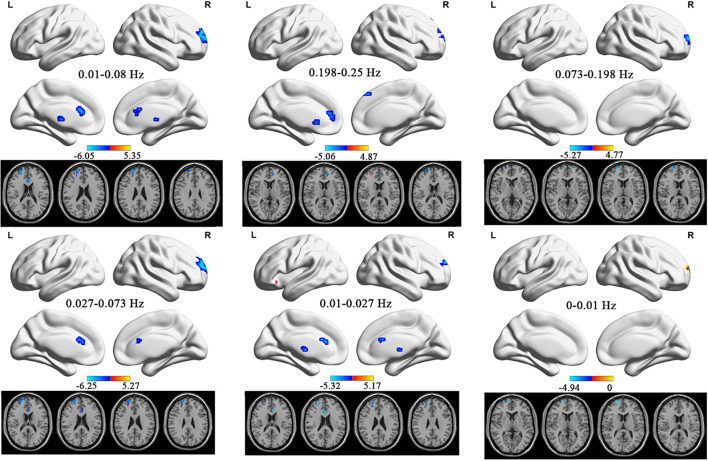
The ALFF differences in the six frequency bands (conventional band, 0.01–0.08 Hz; slow-2, 0.198–0.25 Hz; slow-3, 0.073–0.198 Hz; slow-4, 0.027–0.073 Hz; slow-5: 0.01–0.027 Hz) between TTH patients and HCs. ALFF, amplitude of low-frequency fluctuation; TTH, tension-type headache; HC, healthy control.

In the conventional frequency band, ALFF values significantly increased in the right superior parietal gyrus. They decreased in the left anterior cingulate and paracingulate gyri, left thalamus, and right superior frontal gyrus in TTH patients (see Table 2 and Figure 1). In the slow-2 band, the TTH group exhibited significantly lower ALFF values in the left caudate nucleus, left anterior cingulate and paracingulate gyri, right superior frontal gyrus, left superior frontal gyrus, and right medial superior frontal gyrus (see Table 2 and Figure 1). In the slow-3 band, a significantly lower ALFF (TTH < HCs) in the right superior frontal gyrus was observed (see Table 2 and Figure 1). In the slow-4 band, ALFF values significantly increased in the right superior parietal gyrus. They decreased in the right superior frontal gyrus, left anterior cingulate, and paracingulate gyri in the TTH group (see Table 2 and Figure 1). In the slow-5 band, ALFF values significantly increased in the right superior parietal gyrus and left orbital inferior frontal gyrus. They decreased in the left thalamus, left anterior cingulum angular, and right middle frontal gyrus in the TTH group (see Table 2 and Figure 1). In the slow-6, ALFF values significantly decreased in the right superior frontal gyrus (see Table 2 and Figure 1).

**TABLE 2:** The ALFF difference in each frequency band between TTH and HC.

Brain region	Cluster size (voxel)	Coordinate (x, y, z)	Peak *t* value
**Conventional frequency band (0.01–0.08 Hz)**			
Left anterior cingulate and paracingulate gyri	22	0, 21, 21	−5.2784
Left thalamus	15	−6, −18, 3	−5.0896
Right superior frontal gyrus	89	21, 54, 21	−6.0496
Right superior parietal gyrus	12	30, −69, 60	5.353
**Slow-2 band (0.198–0.25 Hz)**			
Left caudate nucleus	21	−6, 18, −3	−5.0628
Left anterior cingulate and paracingulate gyri	14	−6, 42, 12	−5.0003
Right superior frontal gyrus	12	18, 63, 18	−4.4065
Left superior frontal gyrus	10	21, 60, 27	−4.9852
Right medial superior frontal gyrus	10	6, 33, 48	−4.2998
**Slow-3 band (0.073–0.198 Hz)**			
Right superior frontal gyrus	26	18, 63, 12	−5.27
**Slow-4 band (0.027–0.073 Hz)**			
Right superior frontal gyrus	81	21, 54, 21	−6.2502
Left anterior cingulate and paracingulate gyri	16	0, 21, 21	−5.0099
Right superior parietal gyrus	11	30, −69, 60	5.272
**Slow-5 band (0.01–0.027 Hz)**			
Left orbital inferior frontal gyrus	13	−42, 24, −9	5.1674
Left thalamus	13	−3, −12, 3	−4.6495
Left anterior cingulate and paracingulate gyri	15	−3, 24, 18	−5.0627
Right middle frontal gyrus	22	24, 45, 21	−5.3248
Right superior parietal gyrus	11	30, −69, 60	4.956
**Slow-6 (0–0.01 Hz)**			
Right superior frontal gyrus	13	18, 63, 18	−4.9358

*ALFF, amplitude of low-frequency fluctuation; TTH, tension-type headache; HC, healthy control.*

### Correlation Analysis

**FIGURE 2:**
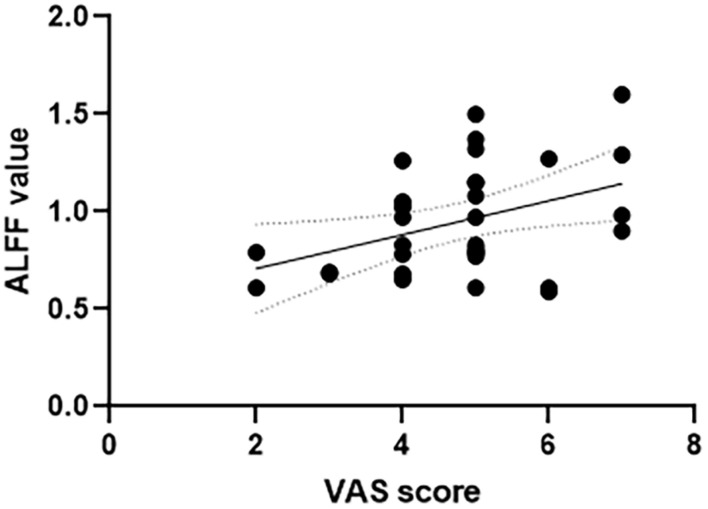
Correlation between VAS scores and ALFF value of the left anterior cingulate and paracingulate gyri in the slow-4 frequency band of 0.027–0.073 Hz. VAS, Visual Analog Scale; ALFF, amplitude of low-frequency fluctuation.

**FIGURE 3:**
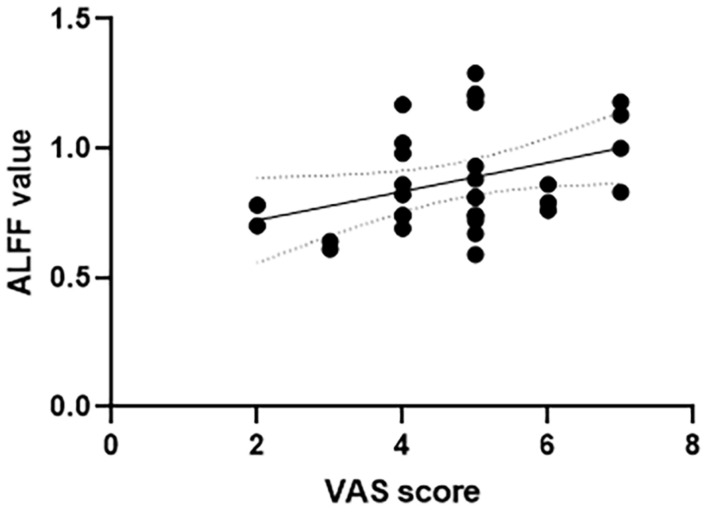
Correlation between VAS scores and ALFF value of the right superior parietal gyrus in the slow-4 frequency band of 0.027–0.073 Hz. VAS, Visual Analog Scale; ALFF, amplitude of low-frequency fluctuation.

The correlation analysis between the ALFF value and VAS scores was conducted in all the six frequency bands. In the slow-4 frequency band of 0.027–0.073 Hz, both the left anterior cingulate and paracingulate gyri and right superior parietal gyrus showed positive correlation with the VAS score (left anterior cingulate and paracingulate gyri: *r* = 0.4, *p* = 0.023; right superior parietal gyrus: *r* = 0.360, *p* = 0.043, see Figures 2, 3). No correlation was found in other five frequency bands (see Supplementary Material for details).

## Discussion

In the current study, we investigated the spontaneous low-frequency fluctuations (LFFs) in TTH patients at rest during six different frequency bands (conventional, 0.01–0.08 Hz; slow-2, 0.198–0.25 Hz; slow-3, 0.073–0.198 Hz; slow-4, 0.027–0.073 Hz; slow-5, 0.01–0.27 Hz; and slow-6, 0–0.01 Hz). Several regions exhibited significant differences in ALFF values in all the six frequency bands. These results indicate that the alterations in regional activity in TTH patients are frequency dependent.

Amplitude of low-frequency fluctuation is an effective method for detecting regional signal alterations of spontaneous activity (Zou et al., 2008). Biswal proposed that the LFFs contained physiologically meaningful information (Biswal et al., 1995). The alterations of ALFF showed the spontaneous neural activity of the brain (Zuo et al., 2010). The enhancement of ALFF shows that the excitability of brain area was increased, and the BOLD signal deviated from the baseline. The weakening of ALFF indicates that neurons are inhibited and that their activity decreases (Huang et al., 2010; Zhang et al., 2010). In the present study, the ALFF method was utilized to measure the low-frequency oscillations (LFOs) in TTH patients.

In most frequency bands, the study revealed that TTH patients showed decreased ALFF values in the middle frontal gyrus and superior frontal gyrus, modulating cortical and subcortical nociceptive pathways (Peyron et al., 1999; Bantick et al., 2002). Similarly, Wang et al. (2014) demonstrated that TTH patients exhibited lower ReHo values in the middle frontal gyrus and superior frontal gyrus. Besides functional changes, Yang et al. (2013) found GM volume reductions in the two areas—the middle frontal cortex is involved in anticipating pain (Ploghaus et al., 1999) and assumed to modulate pain (Kanda et al., 2003). Pain is a subjective sensation, and therefore, its perception is influenced by many cognitive processes such as focus of attention and cognitive modulations of pain (Wang et al., 2016a). The frontal lobe, vital in planning complex cognitive behavior, is highly associated with migraines (Terkelsen et al., 2004; Quevedo and Coghill, 2007; Yang and Raine, 2009; Wang et al., 2016a). Our study adds that it plays a vital role in TTH also. And the decreased ALFF value may indicate that the functional compensation is limited. In addition, the superior parietal gyrus showed increased ALFF in conventional (0.01–0.08 Hz), slow-4 (0.027–0.073 Hz), and slow-5 (0.01–0.027 Hz) frequency bands, which might be a compensatory manifestation. Attention is proposed to influence the perception of pain, and the superior parietal gyrus has been revealed to be related to visuospatial attention (Wu et al., 2016). Positive correlation between the ALFF value and the superior parietal gyrus supported this result. The stronger the spontaneous activity in the superior parietal gyrus, the higher the degree of pain.

Meanwhile, the caudate is an essential component of the basal ganglia, which regulates pain sensation, analgesic responses, and pain signal transmission (Chudler and Dong, 1995; Apkarian et al., 2005). The involvement of the basal ganglia in many aspects of the pain process was supported by preclinical and clinical data (Barker, 1988; Chudler and Dong, 1995). Both external and internal neural drive inputs can be processed by the basal ganglia (Borsook et al., 2010). In addition, the basal ganglia integrate information between the cortical and thalamic regions, and the dysfunctional cortico-basal ganglia-thalamic loops may help maintain chronic pain.

It is worth noting that the thalamus, another critical region of the cortico-basal ganglia-thalamic loops, showed ALFF values altered in the present study (Borsook et al., 2010). It hints that TTH patients were involved in abnormal activities. A lower ALFF value in the thalamus indicated the decreased spontaneous activity, which reflects the pathological damage in the thalamus. Valenzuela-Fuenzalida et al. (2021) conducted a systematic review and found the important role of the thalamus in the migraine. It proposed that the thalamus is involved in the perception of allodynia and is also associated with the pain modulation. In the process of migraine, there are ascending/descending pain pathways between the posterior thalamus and the cortical regions that showed a dysfunction in the modulation of pain (Wang et al., 2016b,c). The present study may support the evidence for the role of the thalamus in the TTH patients.

The anterior cingulate cortex also belongs to basal ganglia-thalamic-cortical loops (Wang et al., 2014). The role of the anterior cingulate cortex in the pain has been well explained. Many neuroimaging studies have declared that the anterior cingulate cortex is a cortical region in pain perception (Talbot et al., 1991; Casey et al., 1994; Coghill et al., 1994; Xu et al., 1997). Early in 1997, Davis et al. demonstrated that pain as well as attention could activate the anterior cingulate cortex using the functional MRI technique (Davis et al., 1997). Moreover, Sawamoto et al. (2000) showed the role of the anterior cingulate cortex in regulating the pain-dependent behavior. The decreased ALFF reflected that the spontaneous neural activity in the anterior cingulate cortex decreased. It may indicate the abnormal function of the anterior cingulate cortex in the TTH patients. However, the correlation analysis showed that ALFF values in the anterior cingulate cortex were positively correlated with VAS score. The weaker the spontaneous activity in the anterior cingulate gyrus, the lower the degree of pain. There was an imbalance between anterior cingulate gyrus nerve activity and clinical manifestations. Since the anterior cingulate gyrus is involved in pain perception, the reduction of pain may be related to the dysfunction of the anterior cingulate gyrus. In the present study, the ALFF in the TTH patients decreased in the left caudate nucleus in the slow-2 band but showed no significantly alterations in other five frequency bands. This finding suggests a frequency specific alternating characterization of intrinsic brain activity. The decreased ALFF value indicated the limited functional compensation and the abnormal functions in the left caudate. Slow-2 frequency band may be sensitive in noticing the abnormalities of the spontaneous brain activity.

Slow-4 (0.027–0.073 Hz) band was found to have better test–retest reliability for measuring LFFs, and Li et al. (2014) declared that slow-4 generated higher ALFF values instead of slow-5 (0.01–0.027 Hz). However, in the current study, slow-4 did not show any specific pattern of intrinsic brain activity. On the contrary, the increased ALFF was found in the right inferior frontal orbital gyrus in slow-5, which reflected the functional compensation of the right inferior frontal orbital gyrus. TTH patients show a high frequency of depression. Patients with frequent headaches have been found more vulnerable to TTH under the influence of depression (Janke et al., 2004). The reduced GM in the right inferior frontal orbital was found in previous studies that explored patients with depression (Yang et al., 2020). This provides strong evidence for the relation between the TTH and depression.

Results with and without age regression were compared. It is obvious that after regression with age as a covariate, the increased ALFF value in angular gyrus in conventional frequency band of 0.01–0.08 Hz, slow-4 frequency band of 0.027–0.073 Hz, slow-5 frequency band of 0.01–0.027 Hz, and slow-6 frequency band of 0–0.01 Hz disappeared. It seems that the angular gyrus is the area most affected by age factor. According to previous study, angular gyrus is involved in cognition and emotion (Grabner et al., 2013). And Hirst et al. (2021) emphasized the importance of angular gyrus in the perception and cognition. Considering that pain is easily affected by perception and cognition (Wang et al., 2016a), the impact of age on patients with tension pain is understandable. Studies have revealed that migraine prevalence is related to age. However, it is more variable for the prevalence of TTH (Straube and Andreou, 2019). The prevalence peaks between the age of 30–39 and decreased slightly (Bendtsen et al., 2010a). In the present study, the age of the TTH group is significantly higher than that of the HC group. However, age does not have much effect on the spatial pattern of results. Since the age span of our subjects is small, further research is needed to extend this conclusion to the whole age group.

Although the findings of our study are inspiring, some limitations deserve further investigation in future work. First, the sample size is relatively small, and thus, the statistical power of the results may be affected. Second, like other rs-fMRI studies, the effects of physiological noise cannot be entirely removed at the relatively low sampling rate (TR = 2,000 ms).

## Conclusion

The ALFF analysis deployed in this study revealed the alterations of ALFF values in multiple frequency bands in the TTH patients. And the abnormalities of LFO amplitude in TTH were frequency dependent. Our findings indicate that using multiple frequency bands will help detect the neural changes in TTH.

## Data Availability Statement

The raw data supporting the conclusions of this article will be made available by the authors, without undue reservation.

## Ethics Statement

The studies involving human participants were reviewed and approved by the Affiliated Hospital of Weifang Medical University Committee on Human Research. The patients/participants provided their written informed consent to participate in this study. Written informed consent was obtained from the individual(s) for the publication of any potentially identifiable images or data included in this article.

## Author Contributions

JR and X-ZJ designed the study. S-XZ collected the data. XL analyzed the data. M-TL wrote the first draft of the manuscript, which JR, X-ZJ, and CA revised the manuscript and all other authors added their comments. All authors approved the manuscript.

## Conflict of Interest

The authors declare that the research was conducted in the absence of any commercial or financial relationships that could be construed as a potential conflict of interest.

## Publisher’s Note

All claims expressed in this article are solely those of the authors and do not necessarily represent those of their affiliated organizations, or those of the publisher, the editors and the reviewers. Any product that may be evaluated in this article, or claim that may be made by its manufacturer, is not guaranteed or endorsed by the publisher.
