# Supplementary material for: Amplitude of Low-Frequency Fluctuation in Multiple Frequency Bands in Tension-Type Headache Patients: A Resting-State Functional Magnetic Resonance Imaging Study
Source: Front Neurosci. 2021 Oct 25;15:742973. doi: 10.3389/fnins.2021.742973 (PMC8573136; doi:10.3389/fnins.2021.742973)
Supplement: Supplementary file 1 [file Data_Sheet_1.docx]

| **Brain Region** | **Cluster size**  **(voxel)** | **Coordinate**  **(x, y, z)** | **Peak *t* value** |
| --- | --- | --- | --- |
| **Conventional frequency band (0.01-0.08Hz)** | | | |
| Right middle orbital frontal gyrus | 22 | 33, 54, -12 | -5.5502 |
| Left thalamus | 11 | -6, -18, 3 | -4.4961 |
| Right superior frontal gyrus | 161 | 21, 54, 21 | -6.5292 |
| Left anterior cingulate and paracingulate gyri | 16 | 0, 27, 21 | -4.7415 |
| Right medial superior frontal gyrus | 16 | 12, 30, 54 | -5.0078 |
| Right angular gyrus | 43 | 45, -66, 54 | 5.3365 |
| **Slow-2 band (0.198-0.25Hz)** | | | |
| Right middle orbital frontal gyrus | 80 | 30, 48, -12 | -5.0093 |
| Left caudate nucleus | 19 | -6, 18, -3 | -5.2244 |
| Right superior frontal gyrus | 54 | 21, 60, 27 | -5.3893 |
| Right medial superior frontal gyrus | 20 | 6, 33, 48 | -4.6066 |
| Left superior frontal gyrus | 15 | -9, 39, 48 | -5.4355 |
| **Slow-3 band (0.073-0.198Hz)** | | | |
| Right middle orbital gyrus | 20 | 30, 51, -12 | -4.9229 |
| Left caudate nucleus | 10 | -3, 15, 0 | -4.6474 |
| Right superior frontal gyrus | 87 | 18, 63, 12 | -5.7469 |
| Left medial superior frontal gyrus | 17 | -3, 60, 18 | -4.7228 |
| Right medial superior frontal gyrus | 24 | 6, 33, 48 | -4.9618 |
| Right superior parietal gyrus | 26 | 30, -69, 60 | 5.3839 |
| **Slow-4 band (0.027-0.073Hz)** | | | |
| Right middle orbital frontal gyrus | 20 | 33, 54, -12 | -5.6741 |
| Right superior frontal gyrus | 173 | 21, 54, 21 | -6.7289 |
| Left anterior cingulate and paracingulate gyri | 14 | 0, 27, 21 | -4.6572 |
| Right angular gyrus | 35 | 39, -69, 54 | 5.3033 |
| Right medial superior frontal gyrus | 20 | 12, 30, 54 | -5.022 |
| **Slow-5 band (0.01-0.027Hz)** | | | |
| Left calcarine | 10 | -21, -66, 0 | -4.6525 |
| Right superior frontal gyrus | 60 | 21, 51, 18 | -5.3932 |
| Left anterior cingulate and paracingulate gyri | 10 | -3, 24, 18 | -4.5235 |
| Right angular gyrus | 38 | 42, -69, 54 | 5.1137 |
| **Slow-6 (0-0.01Hz)** | | | |
| Right superior frontal gyrus | 30 | 18, 63, 18 | -5.0551 |
| Right angular gyrus | 12 | 45, -69, 48 | 4.7174 |

Amplitude of low-frequency fluctuation (ALFF) in multiple frequency bands in tension-type headache patients: a resting-state functional MRI study

**Table 1. The ALFF difference in each frequency band between TTH and HC** **(without age regression)**

**Table 2. Correlation between ALFF values and VAS scores in all the six frequency bands**

| **Regions** |  | **VAS scores** |
| --- | --- | --- |
| **Conventional frequency band (0.01-0.08 Hz)** |  |  |
| Left anterior cingulate and paracingulate gyri | Pearson correlation (*r*) | 0.049 |
|  | Significance (*p*) | 0.792 |
| Left thalamus | Pearson correlation (*r*) | 0.190 |
|  | Significance (*p*) | 0.298 |
| Right superior frontal gyrus | Pearson correlation (*r*) | -0.199 |
|  | Significance (*p*) | 0.274 |
| Right superior parietal gyrus | Pearson correlation (*r*) | -0.071 |
|  | Significance (*p*) | 0.699 |
| **Slow-2 band (0.198-0.25 Hz)** |  |  |
| Left caudate nucleus | Pearson correlation (*r*) | 0.192 |
|  | Significance (*p*) | 0.293 |
| Left anterior cingulate and paracingulate gyri | Pearson correlation (*r*) | -0.006 |
|  | Significance (*p*) | 0.974 |
| Right superior frontal gyrus | Pearson correlation (*r*) | -0.076 |
|  | Significance (*p*) | 0.679 |
| Left superior frontal gyrus | Pearson correlation (*r*) | -0.207 |
|  | Significance (*p*) | 0.255 |
| Right medial superior frontal gyrus | Pearson correlation (*r*) | -0.032 |
|  | Significance (*p*) | 0.863 |
| **Slow-3 band (0.073-0.198 Hz)** |  |  |
| Right superior frontal gyrus | Pearson correlation (*r*) | -0.142 |
|  | Significance (*p*) | 0.438 |
| **Slow-4 band (0.027- 0.073 Hz)** |  |  |
| Right superior frontal gyrus | Pearson correlation (*r*) | 0.138 |
|  | Significance (*p*) | 0.452 |
| Left anterior cingulate and paracingulate gyri | Pearson correlation (*r*) | 0.400 |
|  | Significance (*p*) | 0.023 |
| Right superior parietal gyrus | Pearson correlation (*r*) | 0.360 |
|  | Significance (*p*) | 0.043 |
| **Slow-5 band (0.01-0.027 Hz)** |  |  |
| Left orbital inferior frontal gyrus | Pearson correlation (*r*) | 0.131 |
|  | Significance (*p*) | 0.473 |
| Left thalamus | Pearson correlation (*r*) | -0.156 |
|  | Significance (*p*) | 0.394 |
| Left anterior cingulate and paracingulate gyri | Pearson correlation (*r*) | 0.161 |
|  | Significance (*p*) | 0.378 |
| Right middle frontal gyrus | Pearson correlation (*r*) | -0.197 |
|  | Significance (*p*) | 0.280 |
| Right superior parietal gyrus | Pearson correlation (*r*) | -0.084 |
|  | Significance (*p*) | 0.649 |
| **Slow-6 (0-0.01 Hz)** |  |  |
| Right superior frontal gyrus | Pearson correlation (*r*) | -0.028 |
|  | Significance (*p*) | 0.879 |


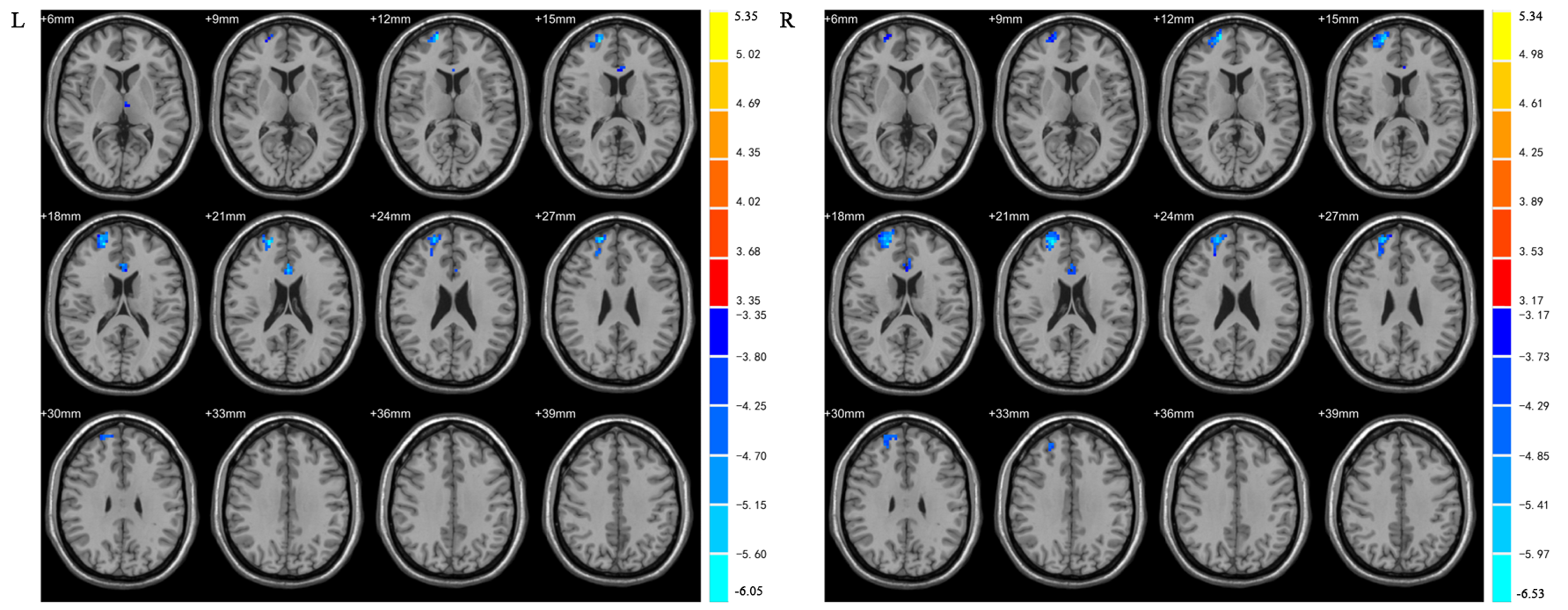


**Figure 1.** Patterns of results that regressed age (L) and patterns of results that didn’t regress age (R) in the conventional frequency band (0.01-0.08 Hz)

**
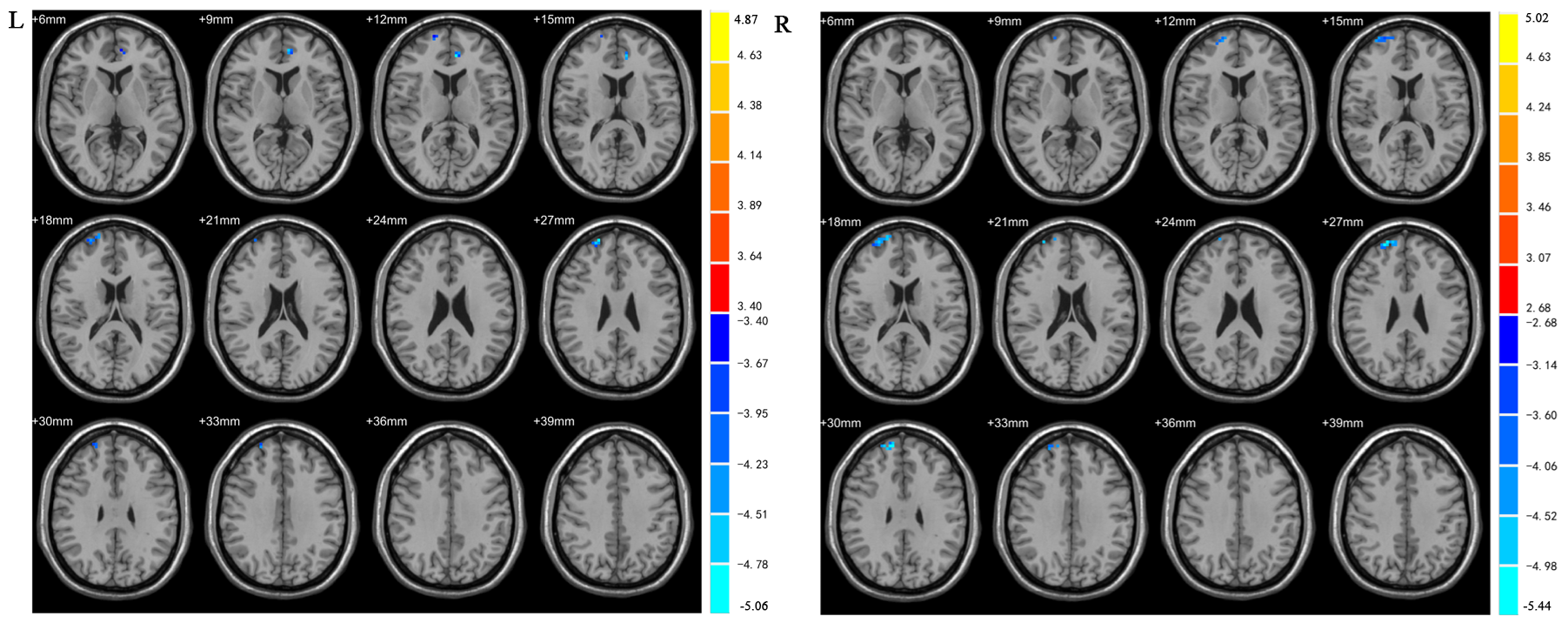
Figure 2.** Patterns of results that regressed age (L) and patterns of results that didn’t regress age (R) in the slow-2 frequency band (0.198-0.25 Hz)

**
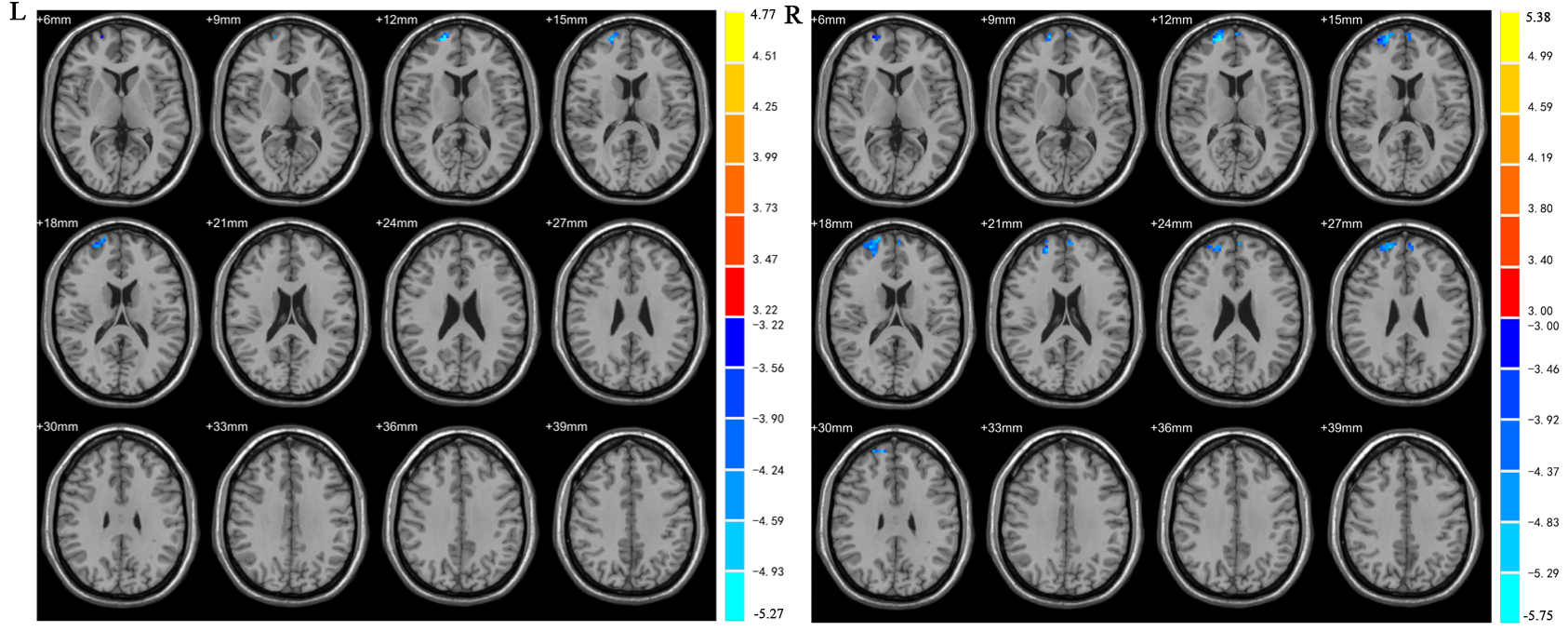
Figure 3.** Patterns of results that regressed age (L) and patterns of results that didn’t regress age (R) in the slow-3 frequency band (0.073-0.198 Hz)


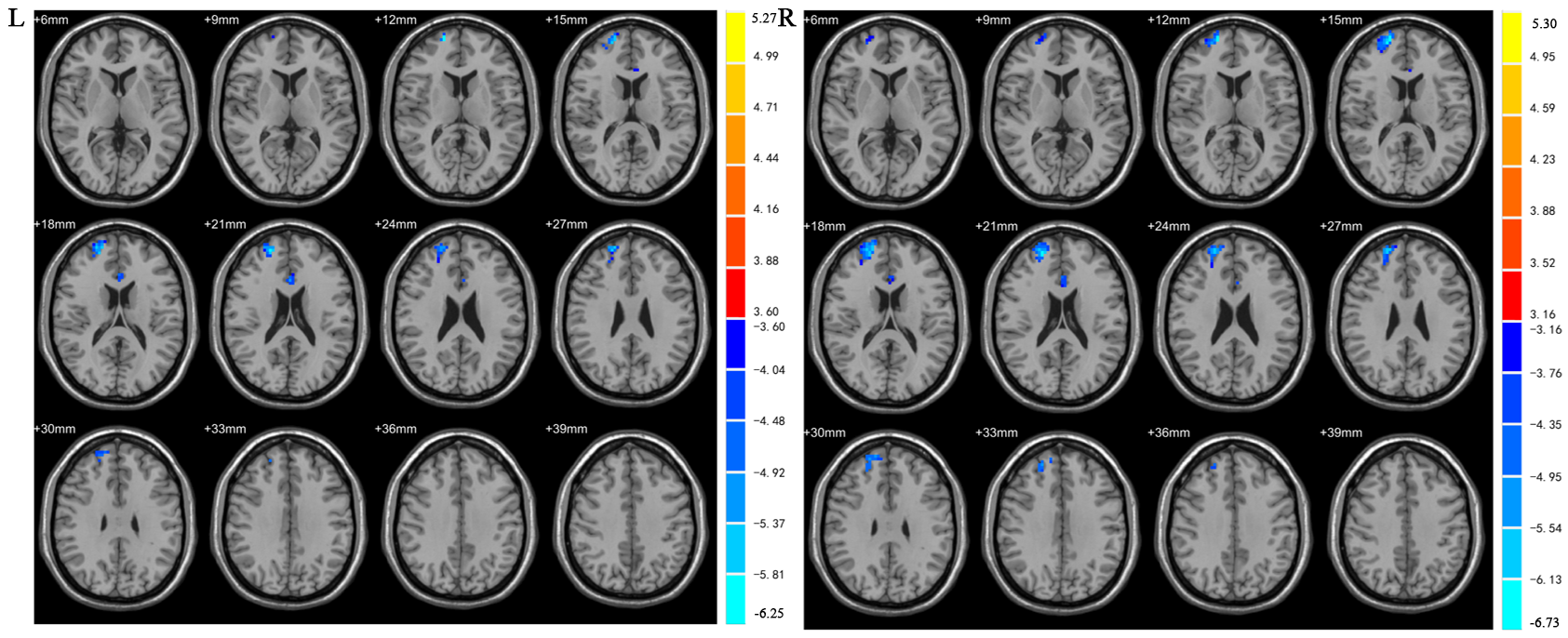


**Figure 4.** Patterns of results that regressed age (L) and patterns of results that didn’t regress age (R) in the slow-4 frequency band (0.027-0.023 Hz)


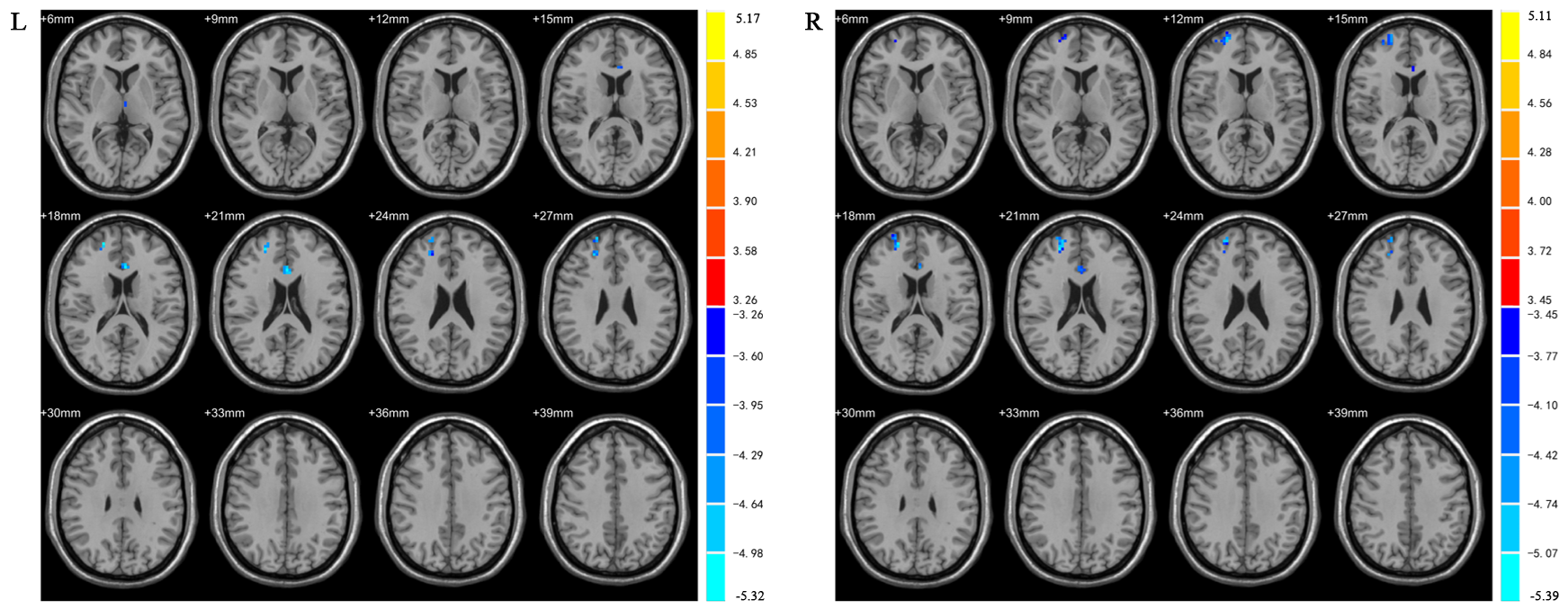


**Figure 5.** Patterns of results that regressed age (L) and patterns of results that didn’t regress age (R) in the slow-5 frequency band (0.01-0.027 Hz)


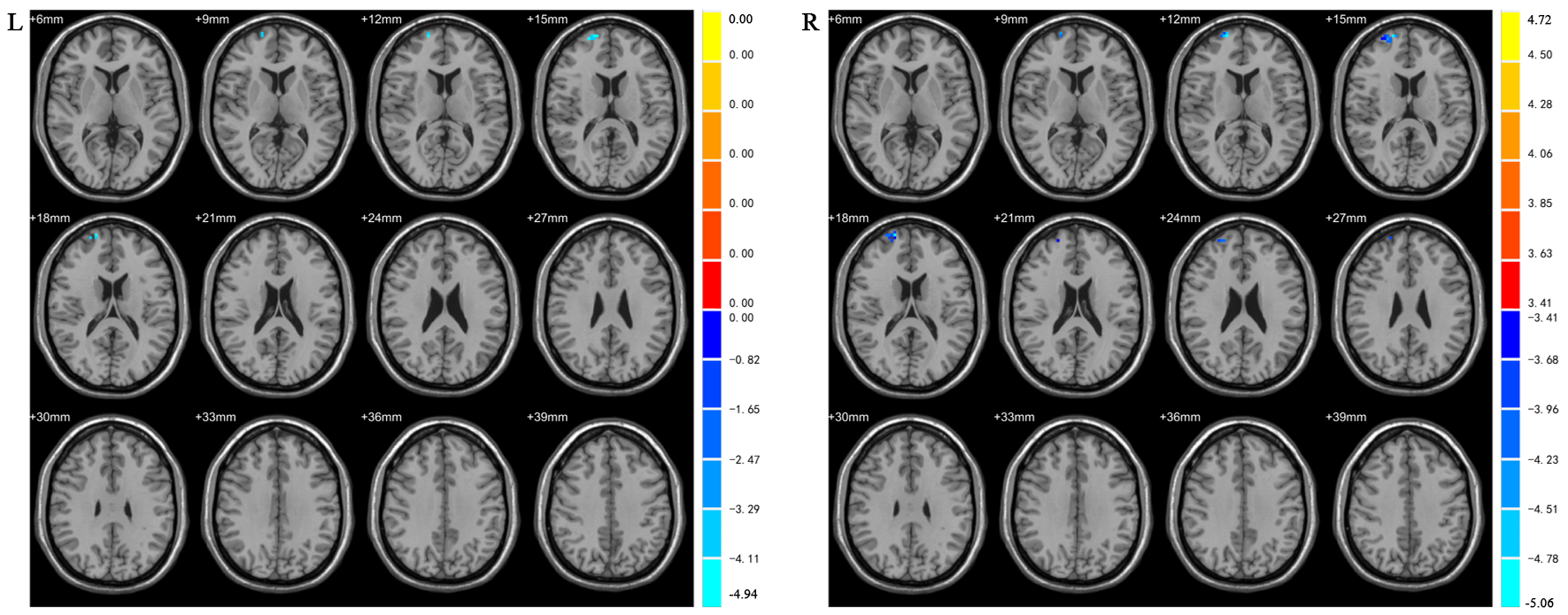


**Figure 6.** Patterns of results that regressed age (L) and patterns of results that didn’t regress age (R) in the slow-3 frequency band (0. -0.01 Hz)
